# Pragmatic randomised trial of a smartphone app (NRT2Quit) to improve effectiveness of nicotine replacement therapy in a quit attempt by improving medication adherence: results of a prematurely terminated study

**DOI:** 10.1186/s13063-019-3645-4

**Published:** 2019-09-02

**Authors:** Aleksandra Herbec, Jamie Brown, Lion Shahab, Robert West, Tobias Raupach

**Affiliations:** 1 Clinical, Educational and Health Psychology, University College London, 1-19 Torrington Place, WC1E 6BT, London, UK; 2 Behavioural Science and Health, University College London, 1-19 Torrington Place, WC1E 6BT, London, UK; 3 Centre for Behaviour Change, University College London, Room 353, 1-19 Torrington Place, London, WC1E 6BT UK; 4 National Centre for Smoking Cessation and Training, 1 Great Western Industrial Centre, Dorchester, DT1 1RD UK; 5 Clinic for Cardiology and Pneumology, University Medical Centre, Universitaetsmedizin Goettingen UBFT, Robert-Koch, Strasse 40, 37075 Goettingen, Germany

**Keywords:** Smoking cessation, Medication adherence, Nicotine replacement therapy, Smartphone application

## Abstract

**Background:**

Nicotine replacement therapy (NRT) bought over the counter (OTC) appears to be largely ineffective for smoking cessation, which may be partially explained by poor adherence. We developed and evaluated the NRT2Quit smartphone app (for iOS) designed to improve quit attempts with OTC NRT by improving adherence to the medications.

**Methods:**

This study was a pragmatic double-blind randomised controlled trial with remote recruitment through leaflets distributed to over 300 UK-based community pharmacies. The study recruited adult daily smokers (≥10 cigarettes per day) who bought NRT, wanted to quit smoking, downloaded NTR2Quit and completed the registration process within the app. Participants were automatically randomly assigned within the app to the intervention (full) version of NRT2Quit or to its control (minimal) versions. The primary outcome was biochemically verified 4-week abstinence assessed at 8-week follow-up using Russell Standard criteria and intention to treat. Bayes factors were calculated for the cessation outcome. Secondary outcomes were self-reported abstinence, NRT use, app use and satisfaction with the app.

**Results:**

The study under-recruited. Only 41 participants (3.5% of the target sample) were randomly assigned to NRT2Quit (*n* = 16) or the control (*n* = 25) app versions between March 2015 and September 2016. The follow-up rate was 51.2%. The intervention participants had numerically higher biochemically verified quit rates (25.0% versus 8.0%, *P* = 0.19, odds ratio = 3.83, 0.61–24.02). The calculated Bayes factor, 1.92, showed that the data were insensitive to test for the hypothesis that the intervention app version aided cessation. The intervention participants had higher median logins (2.5 versus 0, *P* = 0.01) and were more likely to use NRT at follow-up (100.0% versus 28.6%, *P* = 0.03) and recommend NRT2Quit to others (100.0% versus 28.6%, *P* = 0.01).

**Conclusions:**

Despite very low recruitment, there was preliminary but inconclusive evidence that NRT2Quit may improve short-term abstinence and adherence among smokers using NRT. Well-powered studies on NRT2Quit are needed, but different recruitment methods will be required to engage smokers through community pharmacies or other channels.

**Trial registration:**

ISRCTN ISRCTN33423896, prospectively registered on 22 March 2015.

**Electronic supplementary material:**

The online version of this article (10.1186/s13063-019-3645-4) contains supplementary material, which is available to authorized users.

## Background

High-quality evidence from randomised trials shows that nicotine replacement therapy (NRT) is effective when provided with at least some professional support [56]. However, large-scale surveys and prospective studies have found that smokers who buy NRT over the counter (OTC NRT) and do not receive any processional support have quit rates that are similar to, or lower than, those of smokers who quit unaided, even when a range of potential confounding variables are adjusted for [33–35]. One explanation for the discrepancy in effectiveness between NRT in trials and NRT bought OTC is low adherence [3, 5, 15, 19, 31, 48, 55]. There is some research to suggest that better adherence is associated with better cessation outcomes [18, 40, 48, 53]. Smartphone applications (apps) could improve NRT adherence and thus success rates in smokers, especially among those using OTC NRT [36, 47, 49].

Smartphone apps have become an increasingly popular medium to deliver support for a range of health conditions [28] and for medication use [2, 45, 52]. Apps have also been developed to promote smoking cessation, but many of these have been shown to offer only limited support with quitting, and as yet none has been developed specifically to promote NRT adherence [1, 2, 29, 59].

We developed an app for iOS phones, called NRT2Quit, that aimed to support smokers who are using NRT to quit smoking, and the focus was on those who purchase OTC NRT. NRT2Quit was developed following the methods outlined in the Behaviour Change Wheel [41] and was informed by the COM-B model (Capability, Opportunity, Behaviour [32, 43]) and Theoretical Domains Framework (version 2 [4]) as well as the framework of intentional and non-intentional non-adherence [38], the Necessity Concerns Framework [30], the Compliance and Persistence Framework [17], PRIME Theory of Motivation [62] and best clinical practice identified through consultations with the UK’s National Centre for Smoking Cessation and Training.

NRT2Quit was designed to deliver easily accessible general advice on quitting as well as detailed guidelines about NRT, including instructions on medication use, information addressing intentional and modifiable reasons for poor adherence, such as limited knowledge and concerns [30, 46], and features for monitoring and feedback on NRT use. NRT2Quit delivered 25 behaviour change techniques (BCTs) directly addressing NRT use and 27 BCTs addressing quitting in general [42]; in comparison, 12 BCTs on average were found in apps supporting adherence to other medications [45]. It was expected that NRT2Quit would aid cessation by offering advice, reassurance and encouragement to use NRT according to best clinical practice during a quit attempt.

Choosing the right control conditions for the evaluation of apps remains challenging [44]. It was decided that the most appropriate and realistic comparison to NRT2Quit would be a version of the app that offered a minimum credible intervention [44] by being similar to the intervention in many respects (e.g., the registration flow and design) but providing only limited support. There were two main reasons for this approach. First, from an ethical point of view, it was important to offer at least brief advice to smokers who were interested in using an app to help them quit. Second, the similarities between the two arms increase credibility of the control app, potentially minimising the seeking of alternative apps or support, which likely would have increased attrition from the trial and reduced power to detect an effect [44].

Finally, given that the effectiveness of OTC NRT is low [34, 35], it was important to evaluate NRT2Quit in an OTC setting and with no involvement of the researchers, pharmacists or other healthcare professionals (HCPs). It was judged that promoting the study among community pharmacies would offer the best chance to reach smokers who have just purchased OTC NRT and who might not have received additional support with NRT use.

### Aims

The main aim of this pragmatic trial was to evaluate the short-term effectiveness of NRT2Quit. We hypothesised that, in comparison with the control app version, the intervention app would lead to increases in (1) biochemically verified 4-week-long abstinence assessed at 8-week follow-up, (2) self-reported use of NRT, (3) app usage, and (4) satisfaction with the app.

## Materials and methods

### Design

The study was a pragmatic remote two-arm parallel double-blind randomised controlled trial in the UK, and 1:1 automatic randomisation to the intervention and control versions was based on a random numbers function embedded within the registration process within the app, and the study had an 8-week follow-up. The study received ethical approval from the University College London Research Ethics Committee (ID: 5398/001) and was prospectively registered (ISRCTN33423896). The reporting follows Consolidated Standards of Reporting Trials (CONSORT) [51] (Additional file 1) and Template for Intervention Description and Replication (TIDieR) guidelines [25]. Two changes to the protocol were made after the trial began: 7-month follow-up was suspended, and participants using NRT by prescription were included (see Additional file 2 for details). Also, owing to very slow recruitment, the trial was terminated after 18 months.

### Participants

#### Participant recruitment

Recruitment was through self-identification and self-selection and was conducted remotely with no contact with the researcher [24]. The recruitment campaign lasted between 23 March 2015 and 15 September 2016. Recruitment materials were delivered to around 300 UK community pharmacies, mostly through their central managerial offices, with instructions to display and distribute them among smokers who purchase NRT (see Additional file 3 for recruitment materials). The materials directed potential participants to the study website with a detailed study information sheet, information about data processing, end user licence agreement, and links to download the app for free (www.nrt2quit.co.uk). The app could also be found through online searches and on iTunes.

#### Recruitment via community pharmacies

The majority (*n* = 250) of the pharmacies belonged to one large pharmacy chain and were identified through the central managerial office, which was supportive of the study. Fifty more pharmacies were recruited from other major pharmacy chains by communicating with their communications teams and by directly approaching several independent pharmacies. However, no training or direct communication between the researchers and the pharmacy staff was planned (to limit staff burden and to ensure that the context of recruitment of smokers remained as ecological as possible) or possible.

The study promotion could take place only outside of the busy periods, such as Christmas and New Year’s. Only leaflets, rather than larger posters, could be distributed in the participating pharmacies. The leaflet delivery was preceded by internal email communication and accompanied by a printed letter for the head pharmacists, instructing them to place the leaflets near the counters and NRT displays and to provide leaflets to customers purchasing OTC NRT. No professional company was involved in developing the recruitment campaign, and it was not possible to trial the recruitment materials or procedures. Some of the pharmacies in London were visited by the first author to identify ways of improving recruitment, but no further changes to recruitment were possible.

#### Eligibility criteria

Only iPhone users (with iOS 8+) could participate. Eligibility for the trial was assessed on the basis of the information provided during registration via the app: (a) UK-based; (b) age of at least 18 years; (c) daily smoking of at least 10 cigarettes per day; (d) use at least one NRT product; (e) downloaded the app to quit; (f) completed registration process, including providing plausible and complete contact details (these were assessed manually by the researcher); and (g) provided consent to participate that also implied no contraindications for NRT use.

#### Sample size

The target sample size was calculated *a priori* to be 1186 participants (with alpha = 0.05, two-tailed) to have 80% power to detect an expected effect size of odds ratio (OR) of 1.7, translating to 5% difference in self-reported abstinence rates at 8-week follow-up (8% in the control and 13% in the intervention). The expected cessation rates for intention to treat were low as it was expected that attrition from the study would be as high as 50% from each group [23]. The expected effects were small but potentially cost-effective [61].

### NRT2Quit platform and intervention and control arms

NRT2Quit intervention and control app versions were delivered through a single NRT2Quit app platform that could be used offline except for changing the quit date or NRT use to ensure data were synchronised with the server. Both versions of NRT2Quit were developed to be automated and standalone interventions. The advice offered was tailored to the type of NRT product used and the quit date (control and intervention) and to dependence level (intervention only, see 2.3.2). The support was offered for up to 2 weeks before the quit date and 8 weeks post-quit date. Detailed information about the NRT2Quit functionality, the different BCTs delivered within the app (25 BCTs targeting adherence to NRT and 27 targeting smoking cessation in general), screenshots, and user journeys of the intervention and control is provided in Additional file 4. The app was not modified during the trial.

#### NRT2Quit – Control (minimal) version

The control version of the app provided only minimal support with quitting and NRT use: (1) setting of a quit date in the next 2 weeks, (2) very brief advice on the use of selected NRT, (3) brief advice on quitting and managing nicotine withdrawal, (4) progress monitoring (days to and since the quit date), (5) a calendar that displays the quit date and the 8-week questionnaire. It also included (6) brief information about the study and the app. Users could (7) change the quit date and the NRT used.

#### NRT2Quit – Intervention (full) version

The intervention version of NRT2Quit offered the same support as the control version and in addition provided (1) more comprehensive information about NRT in general and about each product (e.g., detailed instructions on use and short articles about key misconceptions such as overdosing), (2) an interactive dashboard for monitoring and feedback on NRT use, (3) daily diary on smoking and NRT use followed by tailored feedback, (4) more detailed advice on quitting, (5) daily tips, (6) additional information about the study team and study rationale, and (7) daily reminders to engage with the app. Feedback and advice on NRT use were minimally tailored to dependence levels (heavy smokers were all those who were smoking 11 to 19 cigarettes per day and smoking the first cigarette within 5 min since waking or all those smoking at least 20 cigarettes per day; moderate smokers consisted of everyone else). The app was designed to encourage daily use (e.g., through app reminders and new daily tips). However, in anticipation of high attrition from the app [23] and given the different preferences for usage of digital interventions among smokers [26], the core content and BCTs were delivered immediately following the registration.

### Procedures

After downloading the app, participants were guided through a tunnelled registration process that included a summary of study information sheet and links to the study website with detailed information, provided informed consent and contact details, completed baseline assessment, entered data on the NRT purchased, and set their quit date (Additional file 5). After registering, participants were automatically randomly assigned to the intervention or control versions of the app and were assigned a unique ID. Participants received an email confirming registration with a link to the study website and contact details to the researchers. Duplicate registrations were excluded following a manual check.

The follow-up took place 8 weeks after the registration (18 May 2015 to 22 November 2016) through an online survey as opposed to within the app (in anticipation that participants would delete the app or switch off notifications). The links to the survey were distributed through emails (up to three reminders) that were personalized [11]. Participants failing to complete the survey were contacted over the phone (up to three calls) to assess smoking status only (a longer survey was judged to be not feasible over the phone). Participants self-reporting prolonged abstinence were posted a saliva kit with instructions, a £20 high street gift voucher as reimbursement, and a freepost envelope addressed to the laboratory and were asked to post the samples as soon as possible [11].

Owing to slow recruitment, it was decided in early August 2016 to prepare for termination of the trial. Bayes factors were calculated on the primary outcome on 18 August 2016 (after 39 eligible participants were recruited), but no hypothesis testing or other analyses were performed. Before NRT2Quit was removed from iTunes on 15 September (the current app users could still access it), two additional participants meeting eligibility criteria joined the study and were included in the analyses reported here. All study procedures, including the follow-up for all participants, were conducted blind to study arm allocation.

### Measurements

#### Baseline assessment

The baseline questionnaire assessed socio-demographic characteristics (age, gender, and having education after 16 years of age versus not), smoking and quitting history (items from the Heaviness of Smoking Index [22], when the last quit attempt was made, past use of cessation aids) and reasons for joining the study (to quit smoking/other). Participants also provided information about the NRT type purchased (NRT patch/fast-acting NRT/combination), how they obtained NRT (OTC/on prescription/both) and whether they received any support with NRT use from HCPs (yes/no).

#### Primary outcome

The primary outcome was self-reported 4-week prolonged abstinence assessed at 8-week follow-up and was verified by saliva cotinine levels of less than 15 ng/mL [64] or, among participants reporting using NRT or e-cigarettes, anabasine levels of less than 1 ng/mL [7, 11]. The pre-registered salivary anabasine cutoff value was based on discussions with the processing lab and the information available at the time of trial set-up (2011–2014). However, as the lab has conducted more studies since, it now recommends a lower cutoff value for salivary anabasine of less than 0.2 ng/mL. Results for the lower cutoff are reported in the footnote of Table 2. Participants lost to follow-up were assumed to have resumed smoking, as per intention-to-treat (ITT) principle.

#### Secondary outcomes

Secondary outcomes were (1) the follow-up parameters: follow-up rate, the re-contact channel (survey/phone), and proportion of saliva samples returned. The online survey at 8-week follow-up assessed: (2) total number of cigarettes smoked in the past 4 weeks (none/<5/≥5); (3) adherence to NRT: (i) use of NRT on the follow-up day (yes/no), (ii) weeks NRT was used (<5/≥5 weeks), and (iii) number of days in those weeks NRT was used (every day/not every day); (4) use of other cessation support such as other medications, behavioural support, or self-help support (yes/no); (5) satisfaction: how helpful was NRT2Quit app for (i) quitting smoking and (ii) using NRT (1 = not at all and 5 = extremely helpful), (iii) whether the participant would recommend the app to others wanting to quit (yes/no). Additionally, (6) data on app usage: (i) number of logins and (ii) number of days users logged in on. Owing to the structure of the app database, data on time spent using the app or on accessing individual app features were not saved.

### Data analysis

The primary outcome was analysed by using Fisher’s exact test. Additionally, unadjusted logistic regressions were conducted for the dichotomised cessation outcomes, and ORs and 95% confidence intervals were calculated. In exploratory sensitivity analyses, participants who reporting using only NRT by prescription (*n* = 14) or for whom that data was missing (*n* = 3) were excluded. All other analyses were pre-planned. For smoking outcomes, participants with missing data were assumed to be smoking.

Bayes factors were calculated for the smoking outcomes as they can distinguish between the likelihood of both the null and alternative hypotheses and assess whether the data provide an insensitive test of the hypotheses [12, 20, 21, 63]. Bayes factors were calculated by using an online calculator that is available for free at http://www.lifesci.sussex.ac.uk/home/Zoltan_Dienes/inference/Bayes.htm. We used a uniform H1 distribution with a possible expected effect size between OR = 1 and OR = 3 versus an H0 of OR = 1. In sensitivity analyses, we used a conservative H1 with a half-normal distribution with the mean of the log OR of 0 and the standard deviation corresponding to expected effect sizes of OR = 1.2, OR = 1.7, and OR = 2.5 [50, 63]. This distribution means that plausible values have been represented between zero and twice the effect size, and smaller values are more likely.

Descriptive statistics are presented for baseline and all secondary outcomes. Categorical variables were compared by using Fisher’s exact test, and chi-squared test and linear-by-linear association for ordered categories, and continuous data using independent *t* test or Mann–Whitney *U* test for data that were not normally distributed. Data on app usage were not normally distributed, but both medians (interquartile ranges) and means (standard deviations) are reported to enable comparison with other studies. All tests were two-sided and alpha was set to 5%.

## Results

### Participants

**Fig. 1:**
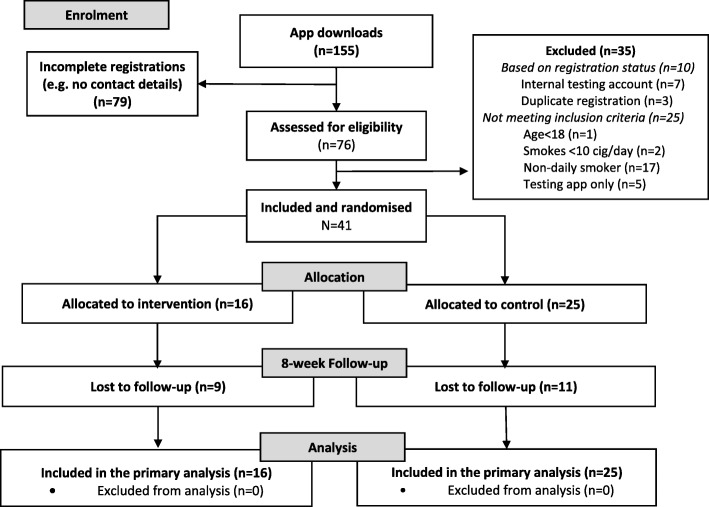
Flowchart of participants in the NRT2Quit trial

Owing to very slow recruitment, the trial was terminated and the analysis involved only 41 participants, who met eligibility criteria for the study, of which 16 (39.0%) were randomly assigned to the intervention app. Figure 1 (based on the CONSORT flow diagram) shows the flow of participants, and Table 1 presents baseline characteristics. A significant minority came across the app through an online search or other channels. About half of participants were female, had education after 16 years of age, and made an attempt to quit in the past 12 months. Almost all participants had used some cessation assistance before; NRT (41.5%) and e-cigarettes (24.4%) were the most common. At baseline, 43.9% of participants reported they were using a fast-acting NRT product on its own and 26.8% were using combined NRT. A quarter of participants obtained advice from HCPs on NRT use.

**Table 1:** Baseline characteristics of NRT2Quit trial participants

	Total (*n* = 41)	Intervention (*n* = 16)	Control (*n* = 25)
Female, % (N)	51.2 (21)	37.5 (6)	60.0 (15)
Age in years, mean (SD)	33.4 (10.02)	32.1 (9.07)	34.3 (10.67)
Has post-16 years qualification, % (N)	51.2 (21)	56.3 (9)	48.0 (12)
CPD, mean (SD)	18.7 (6.54)	17.9 (5.39)	19.2 (7.24)
Smokes within 5 min of waking up, % (N)	39.0 (16)	37.5 (6)	40.0 (10)
HSI, mean (SD)	3.2 (1.32)	3.3 (1.24)	3.2 (1.39)
When made a last quit attempt, % (N)			
Past 12 months	48.8 (20)	68.8 (11)	36.0 (9)
>12 months ago	39.0 (16)	18.8 (3)	52.0 (13)
Never	12.2 (5)	12.5 (2)	12.0 (3)
How learned about the app, % (N)			
Pharmacy	56.1 (23)	31.3 (5)	72.0 (18)
App store or Google search	26.8 (11)	37.5 (6)	20.0 (5)
Other	17.1 (7)	31.3 (5)	8.0 (2)
Used any cessation aids in the past^#^, % (N)			
No aids	7.3 (3)	12.5 (2)	4.0 (1)
NRT	41.5 (17)	62.5 (10)	28.0 (7)
Other medications	12.2 (5)	0.0 (0)	20.0 (5)
Stop-smoking services	9.8 (4)	16.0 (4)	0.0 (0)
Apps	2.4 (1)	0.0 (0)	4.0 (1)
E-cigarettes	24.4 (10)	18.8 (3)	28.0 (7)
Other	2.4 (1)	2.4 (1)	0.0 (0)
Type of NRT used at baseline, % (N)			
Patch only	29.3 (12)	37.5 (6)	24.0 (6)
Fast-acting NRT only	43.9 (18)	37.5 (6)	48.0 (12)
Combination of patch and fast-acting NRT	26.8 (11)	25.0 (4)	28.0 (7)
Reasons for selecting NRT^a^, % (N)			
Used it before	40.0 (16)	50.0 (8)	33.3 (8)
Recommendations from an HCP	15.0 (6)	18.8 (3)	12.5 (3)
Other, including wanting to try something new	45.0 (18)	31.3 (5)	54.2 (13)
Obtained advice from HCPs on NRT use^b^	20.5 (8)	20.0 (3)	20.8 (5)
Method of obtaining NRT^b^, % (N)			
OTC only	25.6 (10)	20.0 (3)	29.2 (7)
Rx only	35.9 (14)	33.3 (5)	37.5 (9)
OTC and Rx	38.5 (15)	46.7 (7)	33.3 (8)

*Abbreviations*: *CPD* cigarettes per day, *HCP* healthcare professional, *HSI* heaviness of smoking index [22], *N* number, *NRT* nicotine replacement therapy, *OTC* over the counter, *Rx* by prescription, *SD* standard deviation.

^#^Participants could select multiple answers; ^a^available for 40 participants; ^b^available for 39 participants

### Follow-up

At 8-week follow-up, 51.2% of participants were successfully contacted (43.8% among intervention and 56.0% among control; Table 3). The online follow-up survey that assessed additional secondary outcomes was completed by 12 (29.3%) participants. The rates were similar across study arms.

### Cessation outcomes

Table 2 presents cessation outcomes assessed at 8-week follow-up. In the ITT analysis, abstinence was biochemically verified for 14.6% of trial participants (25.0% among intervention and 8.0% among control; *P* = 0.19). The results changed only minimally when the cutoff of less than 0.2 ng/mL for salivary anabasine was used. Self-reported abstinence was reported by 17.1% of participants (25.0% among intervention versus 12.0% among control; *P* = 0.40). The Bayes factors (B = 1.92) calculated for biochemically verified and self-reported abstinence suggested that the data were insensitive to distinguishing between the null and experimental hypotheses. The results did not change when the analysis was limited to participants who bought at least one of their NRTs OTC (not reported here).

**Table 2:** Cessation outcomes in NRT2Quit trial (smoking status in past 4 weeks assessed at 8-week follow-up)

	Total (*n* = 41)	Intervention (*n* = 16)	Control (*n* = 25)	*P* value*	OR (95% CI)	Bayes factor^a^ uniform (OR of 1 to 3)	Bayes factor^a^ half normal (OR = 1.2, 1.7, 2.5)
Primary cessation outcome (verified)	% (n)	% (n)	% (n)				
Smoking status ITT^1^							
Not smoking	14.6 (6)	25.0 (4)	8.0 (2)	0.19	3.83 (0.61–24.02)	1.92	1.24, 1.70, 1.99
Assumed to be smoking	85.4 (35)	75.0 (12)	92.0 (23)		–	–	–
Secondary outcome (self-reported)		% (n)					
Smoking status ITT							
Not smoking	17.1 (7)	25.0 (4)	12.0 (3)	0.40	2.44 (0.47–12.78)	1.52	1.18, 1.41, 1.43
Assumed to be smoking	82.9 (34)	75.0 (12)	88.0 (22)		–	–	–
Smoking status ITT							
Not smoking	17.1 (7)	25.0 (4)	12.0 (3)	0.12	–	–	–
Smoking <5 cigarettes	2.4 (1)	6.3 (1)	0.0 (0)		–	–	–
Smoking ≥5 cigarettes	31.7 (13)	12.5 (2)	44.0 (11)		–	–	–
Not contacted/assumed to be smoking	488. (20)	56.3 (9)	44.0 (11)		–	–	–

^1^Two subjects self-reporting not smoking had salivary cotinine of more than 100 ng/mL and anabasine levels of 0.2 ng/mL. With a lower cutoff value for salivary anabasine level suggested recently by the processing lab (<0.2 ng/mL), 18.8% of intervention and 4.0% of control participants met criteria for biochemical verification (odds ratio (OR) = 5.54, 95% confidence interval (CI) 0.52–58.76).

*For 2 × 2 analysis, the *P* value reported is for Fisher’s exact test; otherwise, for Pearson chi-squared.

^a^Bayes factor < 1/3 suggests support for the null hypothesis, Bayes factor > 3 suggests support for the experimental hypothesis, and intermediate values suggest that the data are insensitive (Dienes et al., [21], Brown et al., [12]).

*Abbreviation*: *ITT* intention to treat

### NRT use

Among participants who completed the online survey (*n* = 12), adherence rates were relatively high, and the differences between study arms were not statistically significant except for having used NRT on the survey day (100% in the intervention versus 28.6% in the control arm; *P* = 0.03; see Table 3 for details).

**Table 3:** Secondary outcomes in NRT2Quit trial

	Total (*n* = 41)	Intervention (*n* = 16)	Control (*n* = 25)	*P* value^a^
Follow-up status, % (N)				
Successfully contacted at 8 weeks, % (N)	51.2 (21)	43.8 (7)	56.0 (14)	0.53
Follow-up channel, % (N)				
Survey	29.3 (12)	31.3 (5)	28.0 (7)	0.50^b^
Phone	22.0 (9)	12.5 (2)	28.0 (7)	
Not contacted	48.8 (20)	56.3 (9)	44.0 (11)	
Completed the survey on secondary outcomes, % (N)	29.3 (12)	31.3 (5)	28.0 (7)	1.00^b^
Returned saliva samples when invited, % (n/N)	85.7 (6/7)	100.0 (4/4)	66.7 (2/3)	0.43
App usage after initial registration^1^				
Logins, median (IQR)	1.0 (28.0)	2.5 (12.0)	0 (2.0)	0.01*
Mean (SD)	5.1 (11.17)	10.2 (15.82)	1.8 (4.75)	0.05^c^
Logins, % (N)				
0 logins	41.5 (7)	25.0 (4)	52.0 (13)	0.01^d*^
1 login	12.2 (5)	6.3 (1)	16.0 (4)	
2–5 logins	31.7 (13)	3.7 (6)	28.0 (7)	
≥6 logins	14.6 (6)	31.3 (5)	4.0 (1)	
Days logged in, median (IQR)	1.0 (10.0)	1.5 (5.0)	0.0 (1.0)	0.03*
Mean (SD)	2.7 (5.98)	5.1 (8.35)	1.2 (3.18)	0.10^c^
Days logged in, % (N)^2^				
0 days	41.5 (17)	25.0 (4)	52.0 (13)	0.02^d*^
1 day	29.3 (12)	25.0 (4)	32.0 (8)	
2–7 days	19.5 (8)	31.3 (5)	12.0 (3)	
≥8 days	9.8 (4)	18.8 (3)	4.0 (1)	
Follow-up survey responses^3^	(*n* = 12)	(*n* = 5)	(*n* = 7)	
NRT use and other cessation behaviour, % (N)				
Made a serious QA since registering	91.7 (11)	100.0 (5)	85.7 (6)	1.00
Used additional cessation support	83.3 (10)	60.0 (3)	100.0 (7)	0.15
Used NRT in past 8 weeks	83.3 (10)	80.0 (4)	85.7 (6)	1.00
Used NRT on the day of follow-up	58.3 (7)	100.0 (5)	28.6 (2)	0.03*
Used NRT for ≥5 weeks	66.7 (8)	100.0 (5)	42.9 (3)	0.08
Used NRT every day in weeks when NRT used	58.3 (7)	40.0 (2)	71.4 (5)	0.56
App satisfaction				
App helpful for quitting (1–5)^#^, median (IQR)	3.0 (1.0)	3.0 (1.0)	2.0 (2.0)	0.07
App helpful for quitting (1-5)# Mean (SD)	2.6 (.90)	3.2 (.45)	2.14 (.90)	0.04^c*^
App helpful for NRT use (1–5)^#^, median (IQR)	3.0 (3.0)	4.0 (1.0)	2.0 (2.0)	0.02*
App helpful for NRT use (1–5)#, Mean (SD)	2.7 (1.15)	3.6 (.55)	2.0 (1.00)	0.01*
Recommend to others, % (N)	58.3 (7)	100.0 (5)	28.6 (2)	0.01*

^1^: App usage includes data from any new sessions after registration was completed and excludes the time of registration and initial app exploration following the registration; data on usage and logins may be an underestimation as app use during offline use would not synchronise with the study database if the participants did not access the app online on any future occasion; ^2^: not consecutive days; ^3^: data assessed via online survey among 12 respondents; ^**#**^1 = not at all, 5 = extremely

^a^Fisher’s exact test for 2 × 2 and chi-squared for other categorical variables; ^b^owing to small sample size, a considerable proportion of cells in chi-squared analyses had expected count of less than 5; ^c^unequal variance; ^d^linear-by-linear association; * significant at *p*<0.05

*Abbreviations*: *IQR* interquartile range, *N* number, *NRT* nicotine replacement therapy, *QA* quit attempt, *SD* standard deviation

### App usage

App usage (see Table 3 for details) was low and positively skewed in both conditions, but there was an indication that the intervention participants engaged more (e.g., median number of logins: 2.5 versus 0; *P* = 0.01). This is an underestimation, however, as offline use might not have been saved on servers (e.g., if the participants used the app offline for a period but then permanently disengaged with the app).

### Satisfaction

Among the 12 participants who completed the survey (Table 3), the intervention participants gave higher median ratings of the app as being helpful with NRT use (*P* = 0.02). Additionally, all intervention participants, compared with 28.6% among the control participants, stated that they would recommend the app to others (*P* = 0.01).

## Discussion

### General summary

Owing to very challenging recruitment through community pharmacies, the study was terminated with 41 participants. Nevertheless, it resulted in some important insights. First, the full app version (intervention) led to numerically greater self-reported (25.0% versus 12.0%) and biochemically verified (25.0% versus 8.0%) short-term quit rates, although the differences were not statistically significant when assessed using traditional statics (*P* values). The Bayes factors for the primary outcome suggest ‘anecdotal’ evidence that NRT2Quit could aid cessation but they demonstrate that that data were not sensitive to distinguish between experimental and null hypotheses, and more research is needed. Second, the intervention participants had statistically significant greater engagement and satisfaction with the app. On some indicators of NRT use (e.g., duration), there was an indication that intervention participants used more of it. Taken together, these findings suggest that the support offered by NRT2Quit app may aid cessation and warrants an adequately powered study, but establishing a feasible recruitment channel in the real world may be a major challenge.

The cessation rates reported in this study are similar to those found in other research, but biochemical verification of abstinence was rarely conducted in most other trials [8–10]. The findings suggesting greater effectiveness of the intervention version of the NRT2Quit app are all the more encouraging as the control app version already included several evidence-based BCTs that were shown to improve cessation, including goal setting and monitoring [39]. However, it must be acknowledged that since NRT2Quit was a complex intervention offering both generic support with smoking cessation and dedicated support with NRT use, the trial could not identify specific active ingredients that may be driving the effect. Owing to the small sample, it was also not possible to explore predictors of cessation.

In line with other findings from digital cessation interventions, attrition from the study was high and app engagement was relatively low, although the mean number of logins was in line with usage data from other digital interventions [10, 11, 57]. However, NRT2Quit offered access to the core content immediately following the registration and therefore it is possible that participants had accessed relevant advice already during their first visit, which might have been sufficient to optimise NRT use and improve cessation. The slow recruitment and high attrition could be at least partially explained by the lack of contact with the researchers at enrolment and lack of incentives at follow-up data (except as part of the saliva sample collection) [10].

### Low recruitment rate

Despite securing access to more than 300 community pharmacies across the UK and extending the recruitment window, the study seriously under-recruited; only 4% of the target sample were enrolled. There could be several reasons for this. First, relying on recruitment via printed materials distributed in community pharmacies but with no researcher or HCP engagement proved infeasible. The site visits after the study initiation identified further challenges to recruitment in this context: (i) the leaflet displays were not sufficiently prominent; (ii) at pharmacies embedded within larger supermarkets, the OTC NRT was more prominently displayed on the general supermarket floors (managed by different managerial offices) rather than the pharmacy sections that supported the study; (iii) other cessation campaigns were run concurrently in the pharmacies; and (iv) lack of training and no direct involvement of the pharmacy staff might have resulted in the staff’s not being effective or engaged in study promotion or insufficiently knowledgeable about the study and the app.

Second, potential participants might not have considered it as beneficial to access support with medication use, which could help explain the low interest to download NRT2Quit and join the trial. In a follow-up interview study [27] involving a new group of smokers and ex-smokers who used NRT while quitting, it was found that while they viewed NRT2Quit as potentially beneficial, they reported many barriers in terms of limited capability, opportunity and low motivation to engage with any support and information about NRT use (e.g., leaflets, HCPs, and information on how to use the medications).

Third, NRT2Quit was available only on iOS devices and therefore a considerable proportion of smartphone users who have Android phones could not enrol. However, it is unlikely that developing an Android version of NRT2Quit would improve the recruitment. Research shows that iOS and Android users differ on a range of socio-demographic characteristics and on app use. For example, iOS users are more likely to download and use health apps and engage with more content [13, 58]. iOS users also tend be better off financially [54] and thus might have more disposable income to purchase OTC NRT. On the other hand, given that lower socio-economic status is associated with higher smoking rates, future app developments should also include versions compatible with Android devices.

Fourth, recruiting participants into an online and remote trial such as this one required concealing the differences between the two app versions and prevented promoting the features and advice offered within the intervention app, thus likely leading to a less attractive offer in comparison with other commercially available apps to stop smoking. Finally, the trial took place during a phase marked by a decline in NRT popularity and an increase in the popularity of electronic cigarettes, which was reducing an already small pool of potential participants who use NRT to quit [6].

### Study limitations

The response rate to the online survey was low, which limited the availability of data on NRT use and satisfaction. We were also unable to assess adherence to NRT in detail or account for changes to the patterns of use. Owing to the structure of the app database, it was also not possible to assess engagement with individual app components and fidelity of intervention delivery [37]. Additionally, the burden of joining the current trial was higher than that associated with accessing other cessation apps available on the market (e.g., it involved providing contact details and agreeing to follow-up procedures). It is likely that the recruited participants, as well as those who responded to the follow-up, were more motivated than the general population of smokers. Although this should not have impacted the main results (as motivation would have been similar in the control and intervention groups), the findings should be interpreted with caution and their generalisability is limited.

Another limitation is that, if the app did improve quit rates, we cannot be sure that this was through improved NRT adherence. It is possible that it was through more general support for quitting. The sample size was too small to conduct meaningful mediation analysis involving NRT adherence.

### Study strengths

We collected contact details through the app and followed up participants outside of the app and we conducted biochemical validation of self-reported abstinence, which had a good response rate and which was not carried out in other studies (e.g., [8, 60]). The study also enabled us to make important methodological observations about recruitment and engagement of smokers with smartphone-based support for NRT use. Finally, we evaluated the app in a setting that had higher ecological validity than earlier studies, namely one involving no contact with the researchers at enrolment or incentives for app engagement and survey-based follow-up, some of which have been used in other studies [9, 10, 14].

### Future directions

The findings warrant further development of NRT2Quit and a well-powered study. However, it will be necessary to establish better recruitment channels and methods for such a trial, which in the case of community pharmacies may require engaging the pharmacy staff in active recruitment into the trial and possibly offering incentives [16]. Additionally, it would be relevant to evaluate NRT2Quit as part of face-to-face support to establish whether the app could augment cessation and medication use in this context. Moreover, it is possible that actively promoting the benefits of the full NRT2Quit and offering only this version would lead to better uptake among the smokers. Thus, assessing NRT2Quit in a study with a waitlist control or in an observational study is a possible future direction, especially if the recruitment relies on campaigns in social media.

## Conclusions

In a limited evaluation disrupted by extremely poor recruitment, there was inconclusive evidence that the NRTN2Quit smoking cessation app may impact on short-term quit rates and some preliminary evidence that it may impact on medication use, app use, and satisfaction but this would need to be confirmed in definitive studies. Future research will need to implement more effective recruitment strategies.

## Additional files

Additional file 1: CONSORT (Consolidated Standards of Reporting Trials) 2010 checklist of information to include when reporting a randomised trial. (DOCX 41 kb)

Additional file 2: Changes to protocol and rationale. (DOCX 18 kb)

Additional file 3: Recruitment materials. (DOCX 514 kb)

Additional file 4: Functionality and screenshots of NRT2Quit (intervention and control). (DOCX 647 kb)

Additional file 5: Flow of participants through the NRT2Quit app and the trial. (DOCX 189 kb)

## Data Availability

The datasets used or analysed (or both) during this study are available from the corresponding author on reasonable request.

## Abbreviations

app: Application
BCT: Behaviour change technique
CONSORT: Consolidated Standards of Reporting Trials
HCP: Healthcare professional
ITT: Intention-to-treat
NRT: Nicotine replacement therapy
OR: Odds ratio
OTC: Over the counter
