# Supplementary material for: Pragmatic randomised trial of a smartphone app (NRT2Quit) to improve effectiveness of nicotine replacement therapy in a quit attempt by improving medication adherence: results of a prematurely terminated study
Source: Trials. 2019 Sep 2;20:547. doi: 10.1186/s13063-019-3645-4 (PMC6720069; doi:10.1186/s13063-019-3645-4)
Supplement: Supplementary file 2 — Changes to protocol and rationale. (DOCX 18 kb) [file 13063_2019_3645_MOESM2_ESM.docx]

**NRT2Quit Trial Additional file 2**

**Additional file 2 - Changes to protocol and rationale**

1. The original protocol included a 7-month follow-up. Due to slow recruitment it was judged an inappropriate use of resources to follow-up such a small sample.
2. The initial eligibility criteria specified that smokers had to purchase at least one NRT product OTC, as it was assumed that Rx users have already received behavioural support that could impact on cessation NRT use. Among our sample of otherwise eligible participants, almost a third obtained their NRT on prescription. However, there was no indication that Rx users obtained more support with medication use than OTC users from HPCs (OTC only: 30.0%, Rx only: 21.4%, and OTC and RX users: 13.3%, p=.60). Therefore, participants using only Rx NRT were included in the trial, but were excluded in sensitivity analyses (exploratory analysis).
