# Supplementary material for: Pragmatic randomised trial of a smartphone app (NRT2Quit) to improve effectiveness of nicotine replacement therapy in a quit attempt by improving medication adherence: results of a prematurely terminated study
Source: Trials. 2019 Sep 2;20:547. doi: 10.1186/s13063-019-3645-4 (PMC6720069; doi:10.1186/s13063-019-3645-4)

**Additional file 3** – Recruitment materials

**Figure S1**: Leaflets distributed to community pharmacies in the UK


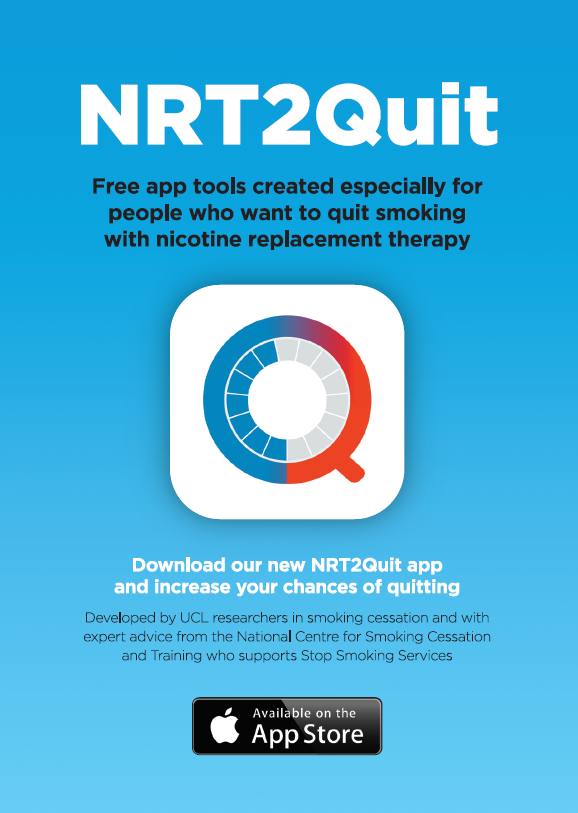

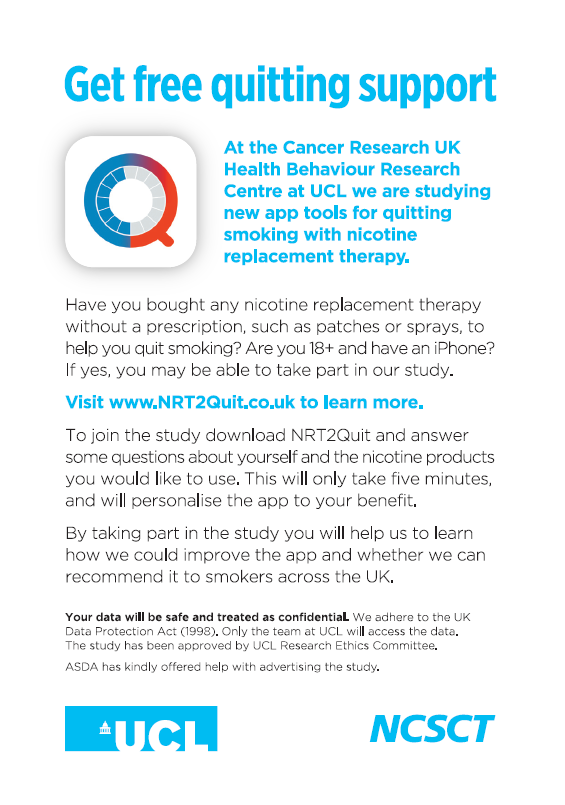


**Figure S2:** Slides with advertisement for display near the till in few of the pharmacies


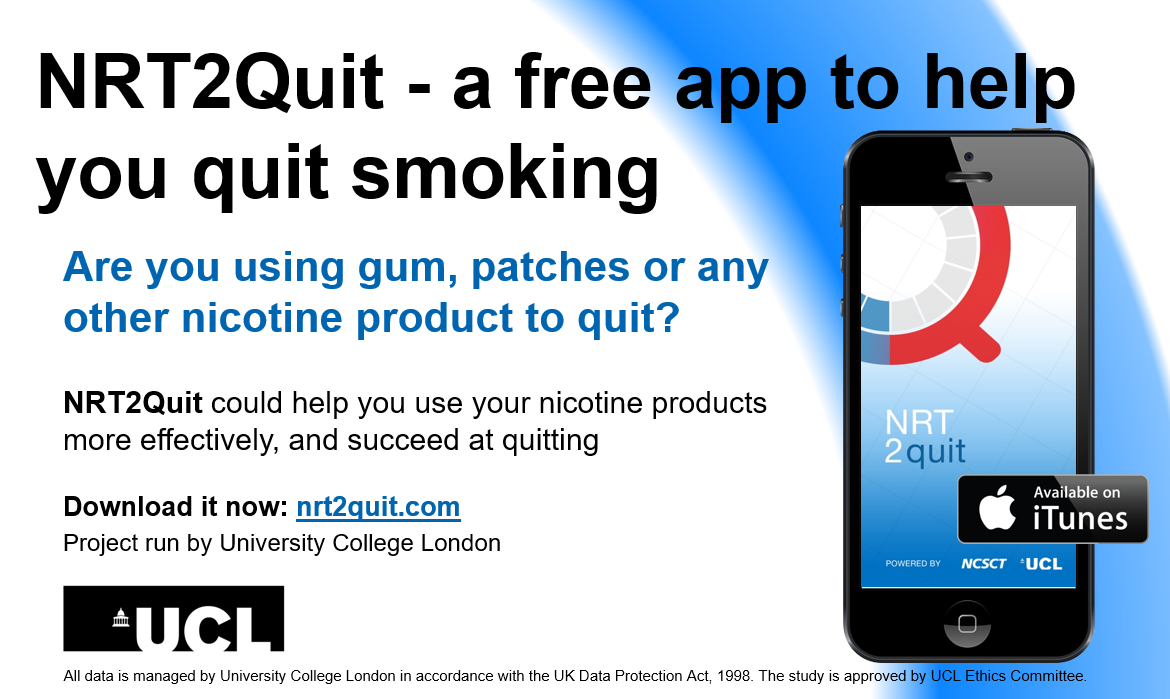

Supplement: Supplementary file 3 — Recruitment materials. (DOCX 514 kb) [file 13063_2019_3645_MOESM3_ESM.docx]
