# Supplementary material for: Pragmatic randomised trial of a smartphone app (NRT2Quit) to improve effectiveness of nicotine replacement therapy in a quit attempt by improving medication adherence: results of a prematurely terminated study
Source: Trials. 2019 Sep 2;20:547. doi: 10.1186/s13063-019-3645-4 (PMC6720069; doi:10.1186/s13063-019-3645-4)
Supplement: Supplementary file 4 — Functionality and screenshots of NRT2Quit (intervention and control). (DOCX 647 kb) [file 13063_2019_3645_MOESM4_ESM.docx]

### **Additional file 4: Functionality and screenshots of NRT2Quit (Intervention and control)**

### **Table S4: Comparison between intervention and control versions of NRT2Quit (greyed out cells = not provided)**

| **Feature and content supporting NRT use** | **Intervention** | | | **Control** | |
| --- | --- | --- | --- | --- | --- |
|  | **BCT Smoking Taxonomy^[[1]](#footnote-1)^** | **BCTs**  **V1 Taxonomy^[[2]](#footnote-2)^** | **BCT Smoking Taxonomy** | | **BCTs**  **V1 Taxonomy** |
| **Registration and Setting of the Quit Date** |  |  |  | |  |
| Setting up of the quit date | BS4 | - 1. 1.3 | BS4 | | 1.1, 1.3 |
| Reassuring feedback on NRT purchased | BM7, RC10 | 2.2, 3.1, 10.4, 15.1 | - | | - |
| Making a pledge for not-smoking and NRT use | BM6 | 1.8 , 13.5, | - | | - |
| Features to update the quit date and NRT use | BS4, RC4 | 1.1, 1.3 | BS4 | | 1.1, 1.3 |
| **Support with NRT Use** |  |  |  | |  |
| Brief advice on NRT use | A1 | 4.1 | - | | - |
| Comprehensive advice on NRT and its use | A1, BM5, RC6 | 4.1, 4.2, 4.3, 4.4, 5.1, 5.3, 5.6, 6.2, 7.1, 8.1, 8.3, 12.1 | - | | - |
| Monitoring and feedback of NRT use (Dashboard, daily diary and additional tailored sessions) | A4, BM7, BS6, RD1, R14 | 1.6, 2.2, 2.3, 3.1, 15.1 | - | | - |
| **General cessation advice and other information** | **-** |  |  | |  |
| Monitoring of smoking status (daily diary) | BS6, R14 | 2.2., 2.3, 2.4, 2.7, 15.1, | - | | - |
| Feedback on smoking status | BM7 | 1.6, 10.4, 15.1 | - | | - |
| Pre- and post-quit daily tips | A2, BM1, BM2, BM8 BM10, BS2, BS3, BS7, BS8, BS10, BS11, RC10, | 5.1, 5.3, 5.5, 5.6, 6.2, 7.1, 8.2, 9.1, 11.1, 11.2, 12.1, 12.2, 12.3, 12.4, 13.5, 15.1, | - | |  |
| Generic advice on quitting | BS2, BM8, BM1, BM2, BM5, RC6 | 4.1, 4.2, 4.3, 5.1, 5.3, 5.6, 6.2, 7.1, 8.2, 9.1, 11.1, 11.2, 12.1, 12.2, 12.3, 12.4, 13.5, 15.1 | BS2*, BM1*, BM2*, BM5*, RC6* | | 5.1*, 6.2*, 10.4*, 11.1* |
| Information on stop smoking medications | A1, RD2 | 5.1, 5.6, 11.1 | - | |  |
| Information about the app, study and the team | RC4 | 9.1 | A1* | | 9.1* |
| **Calendar / Counting days to and from the quit date** |  |  |  | |  |
| Outline of the quit plan and reminders to obtain NRT | BS3 | 12.5 | - | | - |
| Display the date of the follow-up | N/A | N/A | N/A | | N/A |
| Displaying days to and since the quit date | BS3 | 12.5 | BS3 | | 12.5 |
| **Daily reminders to engage with the app and advice** | BS6 | 7.1 | - | | - |

***=brief and simplified**

**Figure S.4a:** Visual summary of the differences in the architecture and functionality of NRT Control vs. Intervention apps.

**
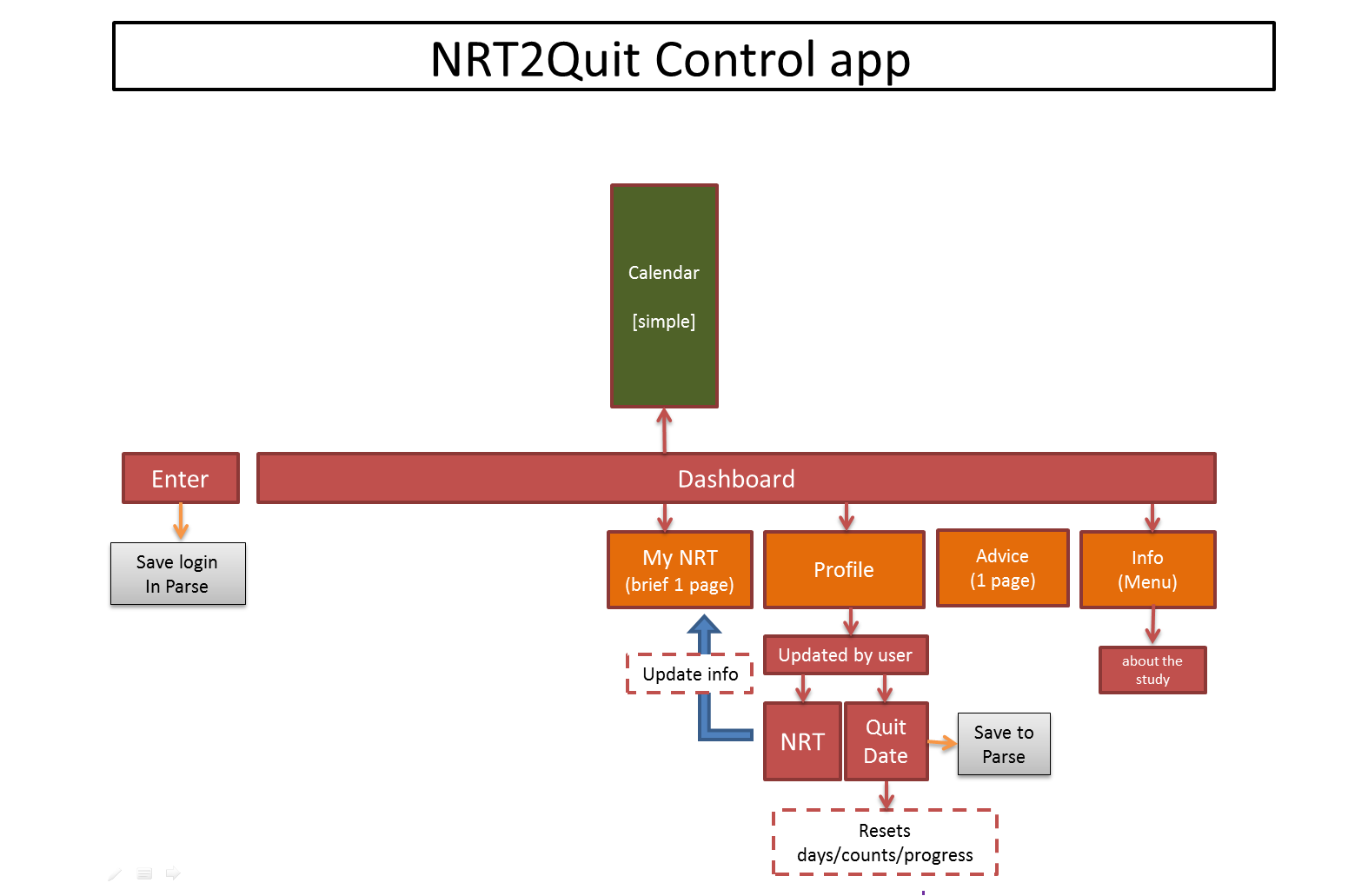

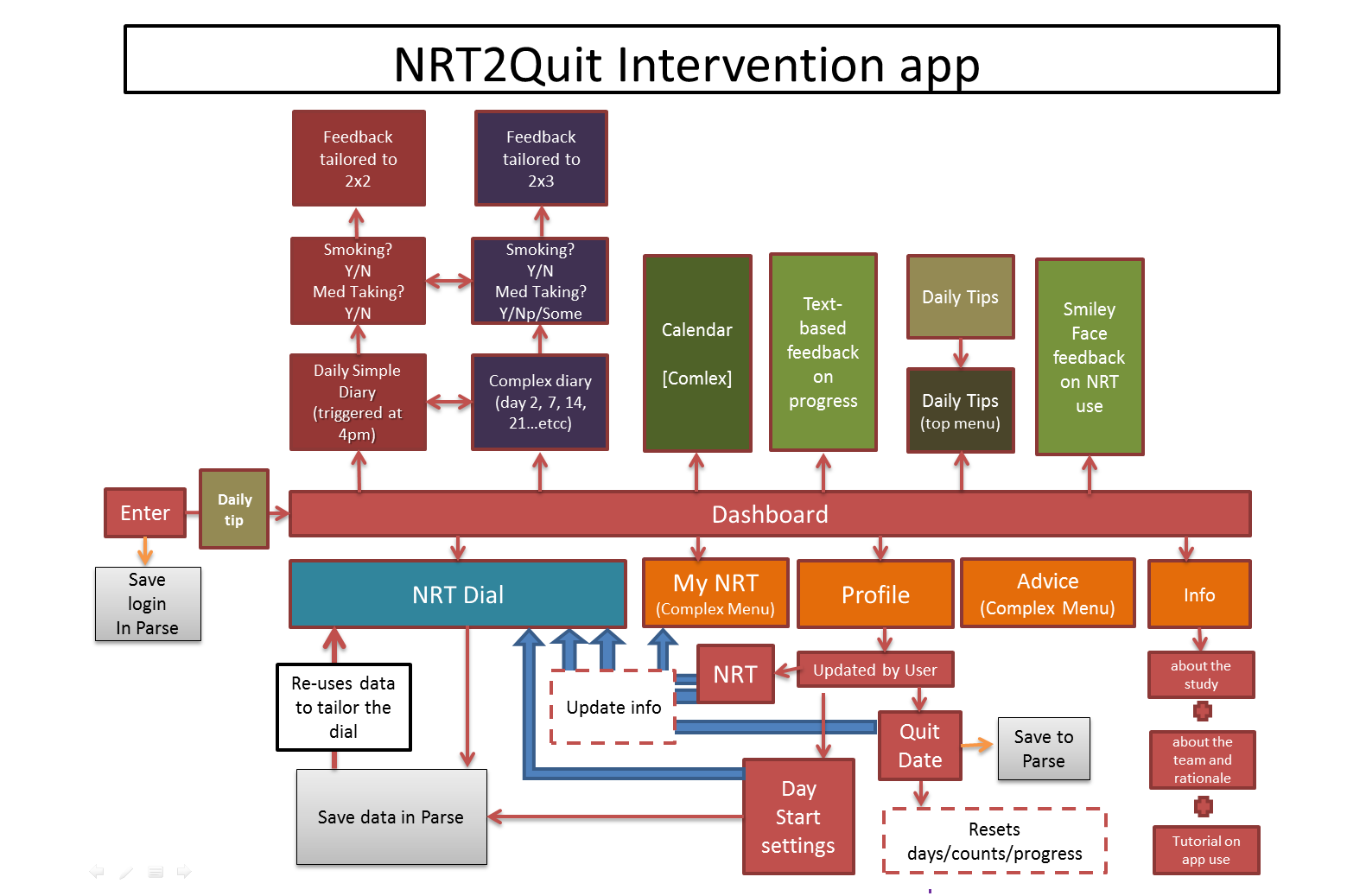
**

###

### **Figure S.4b: Selected Screenshots of NRT2Quit**


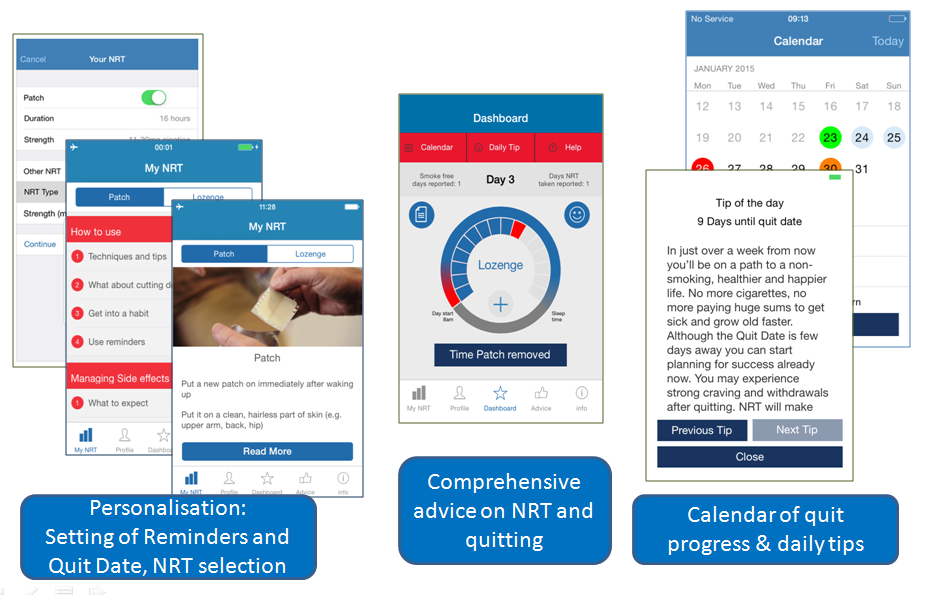


###
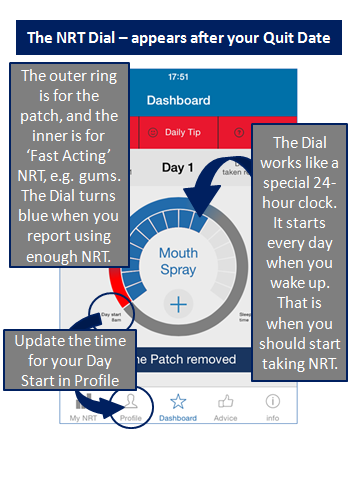

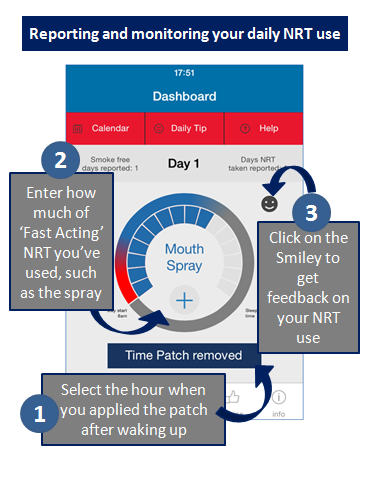

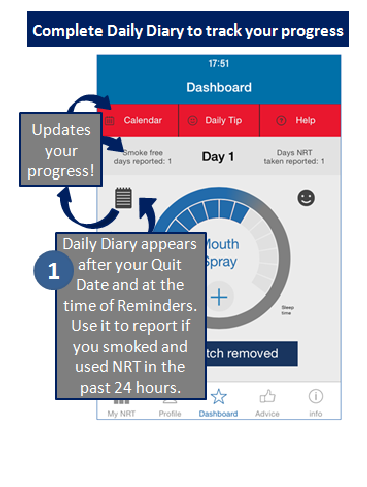

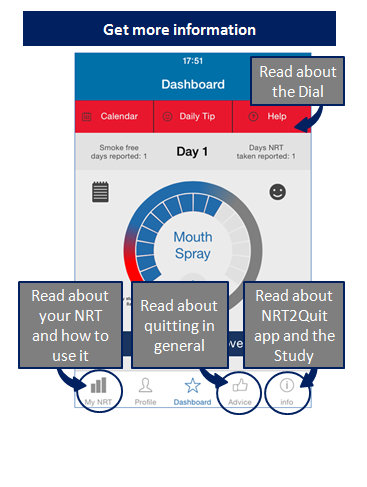

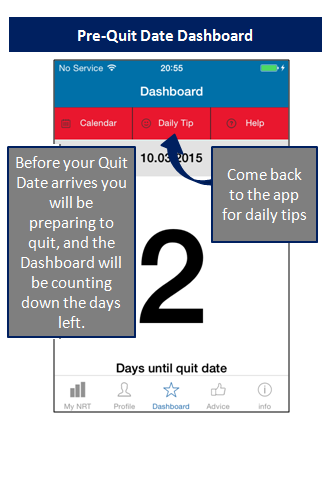
**Figure S.4c:** NRT2Quit – tutorial for Intervention users about NRT2Quit and the main dashboard

1. Michie, S., et al. (2011). Development of a taxonomy of behaviour change techniques used in individual behavioural support for smoking cessation. *Addictive behaviors*, *36*(4), 315-319. [↑](#footnote-ref-1)
2. Michie, S., et al., (2013). The behavior change technique taxonomy (v1) of 93 hierarchically clustered techniques: building an international consensus for the reporting of behavior change interventions. *Annals of behavioral medicine*, *46*(1), 81-95. [↑](#footnote-ref-2)
