# Supplementary material for: Pragmatic randomised trial of a smartphone app (NRT2Quit) to improve effectiveness of nicotine replacement therapy in a quit attempt by improving medication adherence: results of a prematurely terminated study
Source: Trials. 2019 Sep 2;20:547. doi: 10.1186/s13063-019-3645-4 (PMC6720069; doi:10.1186/s13063-019-3645-4)

### **Additional file 5 –** Flow of participants through NRT2Quit app and the trial

### **Figure S6:** Flow of participants through the NRT2Quit app and the trial


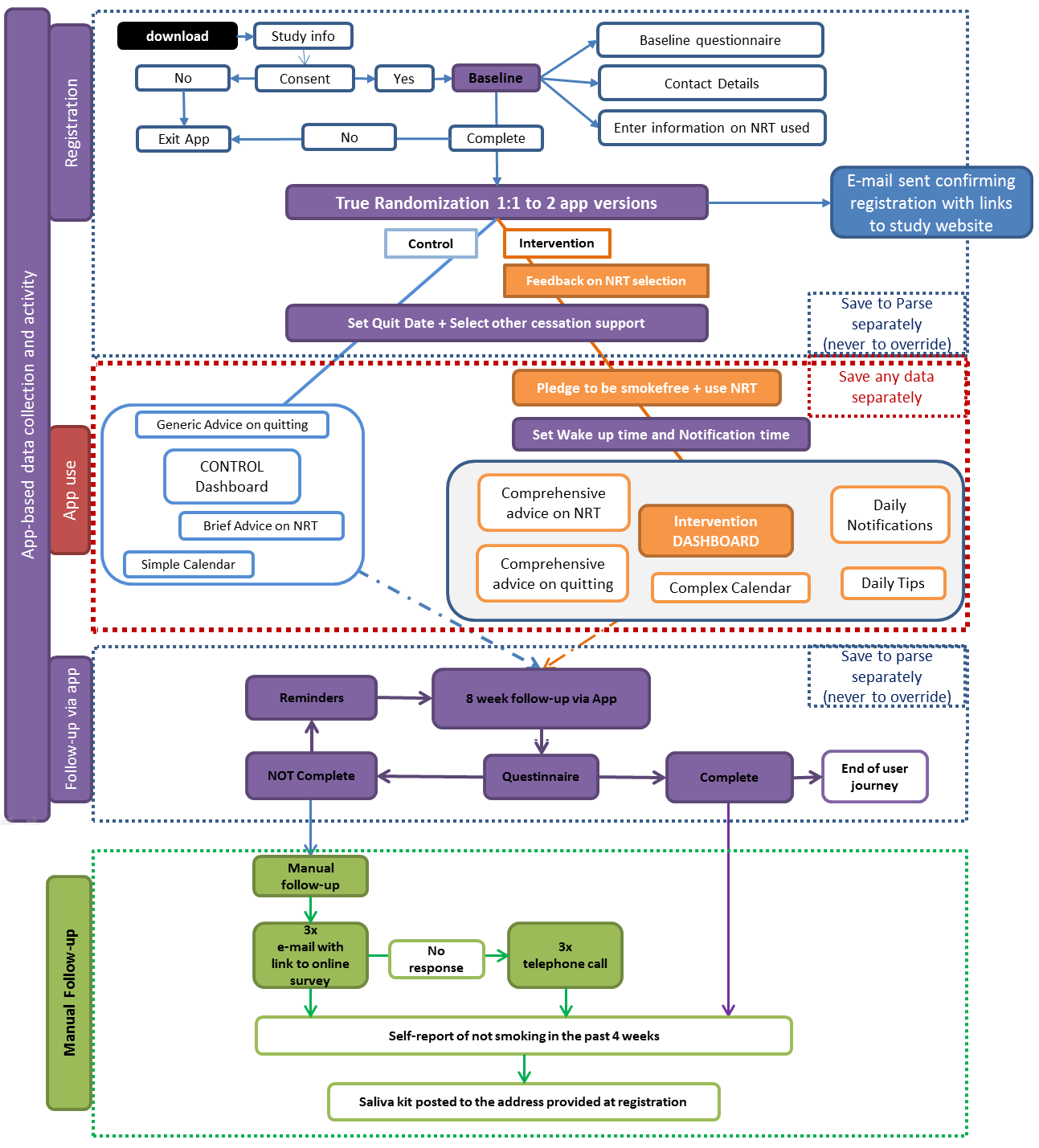

Supplement: Supplementary file 5 — Flow of participants through the NRT2Quit app and the trial. (DOCX 189 kb) [file 13063_2019_3645_MOESM5_ESM.docx]
